# The ancestor of the *Paulinella *chromatophore obtained a carboxysomal operon by horizontal gene transfer from a *Nitrococcus*-like γ-proteobacterium

**DOI:** 10.1186/1471-2148-7-85

**Published:** 2007-06-05

**Authors:** Birger Marin, Eva CM Nowack, Gernot Glöckner, Michael Melkonian

**Affiliations:** 1 Botanisches Institut, Lehrstuhl I, Universität zu Köln, Gyrhofstr. 15, 50931 Köln, Germany; 2 Fritz-Lipmann Institut, Leibniz Institut für Altersforschung, Beutenbergstr. 11, 07745 Jena, Germany

## Abstract

**Background:**

*Paulinella chromatophora *is a freshwater filose amoeba with photosynthetic endosymbionts (chromatophores) of cyanobacterial origin that are closely related to free-living *Prochlorococcus *and *Synechococcus *species (PS-clade). Members of the PS-clade of cyanobacteria contain a proteobacterial form 1A RubisCO (ribulose-1,5-bisphosphate carboxylase/oxygenase) that was acquired by horizontal gene transfer (HGT) of a carboxysomal operon. In rDNA-phylogenies, the *Paulinella *chromatophore diverged basal to the PS-clade, raising the question whether the HGT occurred before or after the split of the chromatophore ancestor.

**Results:**

Phylogenetic analyses of the almost complete rDNA operon with an improved taxon sampling containing most known cyanobacterial lineages recovered the *Paulinella *chromatophore as sister to the complete PS-clade. The sequence of the complete carboxysomal operon of *Paulinella *was determined. Analysis of RubisCO large subunit (*rbcL*) sequences revealed that *Paulinella *shares the proteobacterial form 1A RubisCO with the PS-clade. The γ-proteobacterium *Nitrococcus mobilis *was identified as sister of the *Paulinella *chromatophore and the PS-clade in the RubisCO phylogeny. Gene content and order in the carboxysomal operon correlates well with the RubisCO phylogeny demonstrating that the complete carboxysomal operon was acquired by the common ancestor of the *Paulinella *chromatophore and the PS-clade through HGT. The carboxysomal operon shows a significantly elevated AT content in *Paulinella*, which in the *rbcL *gene is confined to third codon positions. Combined phylogenies using *rbcL *and the rDNA-operon resulted in a nearly fully resolved tree of the PS-clade.

**Conclusion:**

The HGT of the carboxysomal operon predated the divergence of the chromatophore ancestor from the PS-clade. Following HGT and divergence of the chromatophore ancestor, diversification of the PS-clade into at least three subclades occurred. The γ-proteobacterium *Nitrococcus mobilis *represents the closest known relative to the donor of the carboxysomal operon. The isolated position of the *Paulinella *chromatophore in molecular phylogenies as well as its elevated AT content suggests that the *Paulinella *chromatophore has already undergone typical steps in the reductive evolution of an endosymbiont.

## Background

*Paulinella chromatophora *is a thecate filose amoeba of the Rhizaria that contains a photosynthetic entity of cyanobacterial origin termed chromatophore. A similar process initiated the evolution of plastids likely more than a billion years ago. It has previously been shown that neither the *Paulinella *host cell nor the chromatophores are related to the eukaryotic lineage containing primary plastids. Instead, the *Paulinella *chromatophore is affiliated with free-living *Prochlorococcus *and *Synechococcus *spp. (PS-clade), and thus represents the product of a second primary endosymbiosis leading to photoautotrophic eukaryotes [1,2]. It is currently debated whether the *Paulinella *chromatophore represents an organelle comparable to a primary plastid, or merely a stable intracellular symbiont [3-5]. However, the extent of genome reduction as well as the presence or absence of gene transfers and protein import pathways are currently unknown for *Paulinella*, and only three gene cluster (4.3 to 9.4 kb) on the chromatophore genome have been analyzed to date and compared to free-living cyanobacterial relatives [1,6].

Interestingly, the closest relatives of the *Paulinella *chromatophore (the PS-clade) possess a proteobacterial form 1A RubisCO (ribulose-1,5-bisphosphate carboxylase/oxygenase), in contrast to the remaining cyanobacteria and plastids (except rhodoplasts) with the 'typical' form 1B RubisCO [7-9]. The proteobacterial form 1A RubisCO is part of a carboxysomal operon encoding genes for both subunits of RubisCO (*rbcL*, *rbcS *= *cbbL*, *cbbS*) as well as genes for carboxysomal shell proteins and a carboanhydrase [10-12]. Cyanobacteria of the PS-clade may have acquired the complete carboxysomal operon by horizontal gene transfer (HGT) from a proteobacterial donor [10,11]. Carboxysomes containing a form 1A RubisCO are referred to as α-carboxysomes, and thus, the PS-clade has been designated as α-cyanobacteria, in contrast to β-cyanobacteria with a form 1B RubisCO integrated in β-carboxysomes [11].

Previous phylogenies based on rRNA operon sequence data resolved the *Paulinella *chromatophore as sister to marine *Synechococcus *and *Prochlorococcus *spp (α-cyanobacteria) [1]. The intermediate position of *Paulinella*, diverging between α- and β-cyanobacteria, raises the question whether the HGT of the carboxysomal operon occurred before or after the divergence of the ancestor of the *Paulinella *chromatophore, i.e. whether the chromatophore evolved from an α- or β-cyanobacterium. Due to missing data, affiliation to α- or β-cyanobacteria is also unknown for the *Cyanobium*-clade, which, besides marine *Synechococcus *and *Prochlorococcus *clades, represents the third major lineage in the PS-clade (e.g. [13-15]). Analyses of one member of the *Cyanobium*-clade (strain WH 5701) already indicated its individual divergence separate from *Paulinella *and both marine PS-subclades [6].

In this study, we have determined the sequence of the complete carboxysomal operon from the chromatophore of *Paulinella *and a bacterioferritin gene downstream of the carboxysomal operon. Additionally, we determined several *rbcL *and rDNA sequences from other cyanobacteria including the *Cyanobium*-clade. Our data reveal that the *Paulinella *chromatophore as well as the *Cyanobium*-clade display proteobacterial α-carboxysomes, and contain form 1A RubisCO. Furthermore, phylogenies of RubisCO and comparison of gene arrangement types of the carboxysomal operon revealed the γ-proteobacterium *Nitrococcus mobilis *as the closest known relative of the donor in the HGT of the carboxysomal operon. The neighbouring bacterioferritin gene was co-transferred through the same HGT event. Increased AT-content over the carboxysomal operon in *Paulinella *may reflect genomic adaptation to an endosymbiotic lifestyle.

## Results and Discussion

### Phylogenetic analysis of the ribosomal RNA operon

**Figure 1:**
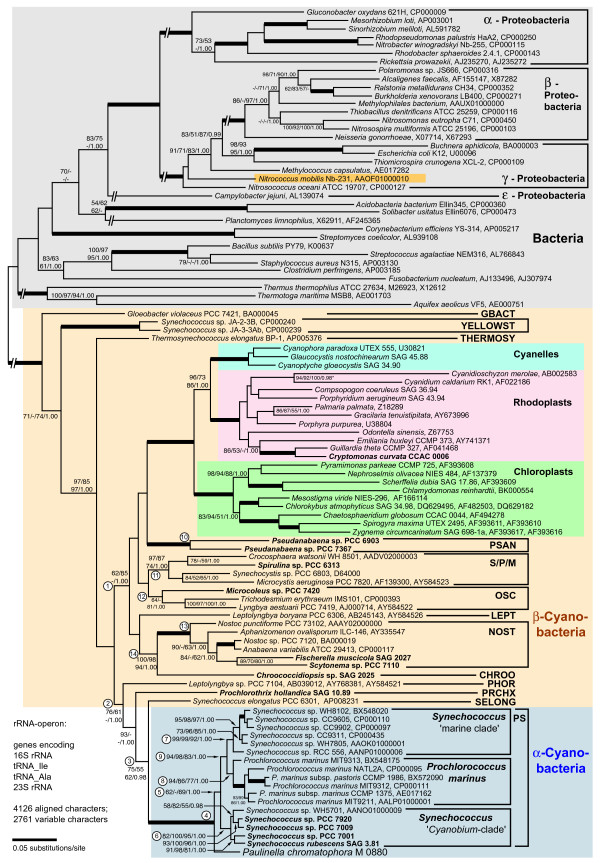
**Phylogenetic position of the *Paulinella *chromatophore within the cyanobacteria inferred by complete rRNA operon sequence comparisons**. The tree topology was generated by maximum likelihood (ML) analyses using the GTR+I+Γ model. The nodal support values are bootstrap values ≥ 50% obtained by ML (100 replicates), neighbor-joining (NJ; GTR+I+Γ model; 1000 repl.), maximum parsimony (MP; 1000 repl.), and Bayesian posterior probabilities (≥ 0.95). Branches in bold have maximal support (100%; 1.00) by all methods; interrupted branches were graphically reduced to 30% of their original length. Taxa in bold were newly determined for this study; strain designations and EMBL/GENBANK accession numbers are also given. Cyanobacterial clade abbreviations: GBACT (*Gloeobacter*), PSAN (*Pseudanabaena*), S/P/M (*Synechocystis*/*Pleurocapsa*/*Microcystis*), OSC (*Oscillatoria*), LEPT (*Leptolyngbya*), NOST (*Nostoc*), and PHOR (*Phormidium*): modified sequence groups after [17]; YELLOST (thermophilic "*Synechococcus*" from Yellowstone NP), THERMOSY (*Thermosynechococcus*), CHROO (*Chroococcidiopsis*), PRCHX (*Prochlorothrix*), SELONG (*Synechococcus elongatus*), and PS (*Prochlorococcus*/*Synechococcus*). Encircled numbers indicate clades/branches that were analyzed by single-gene analyses, and by NJ using the LogDet+I-model (see text and Additional File 1).

One goal of this study was to determine the precise phylogenetic position of the *Paulinella *chromatophore within the cyanobacterial radiation. Cyanobacterial phylogeny is still a challenge since the standard phylogenetic marker, 16S rDNA, is not very informative in global analyses (see below). On the other hand, previous studies using extended data sets, either the complete rDNA operon [1] or several protein coding genes [6,16] suffered from limited taxon sampling. Here, we have extended the taxon sampling of nearly complete rDNA operon sequences to include all major cyanobacterial clades [17,18] with one to several representatives. Therefore, rDNA sequences from one plastid and 12 cyanobacteria were determined (taxa in bold in Figure 1). Together with data from newly released genome projects, the resulting alignment contained sequences from 36 bacteria, 42 cyanobacteria and 23 plastids.

**Figure 2:**
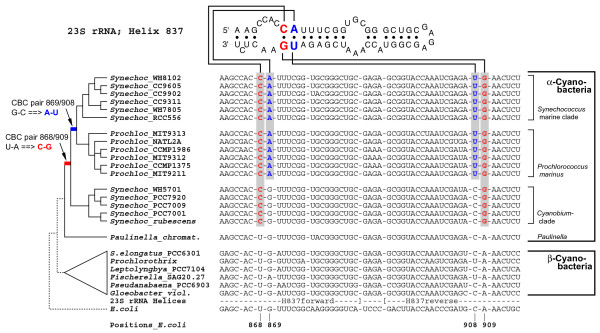
**Synapomorphy support in the 23S rRNA for the sister-group relationship between *Paulinella *and free-living α-cyanobacteria**. Shown is the alignment and secondary structure diagram of Helix 837 in the 23S rDNA, with two RNA base pairs highlighted that represent synapomorphies of α-cyanobacterial clades to the exclusion of *Paulinella *and other prokaryotes. Sequence data and evolutionary changes are plotted on a simplified phylogram (NJ-bootstrap consensus tree). Pair 868/909 shows a uniquely derived CBC (compensatory base change: U-A → C-G) of all free-living α-cyanobacteria; the neighbouring pair 869/908 changed in the common ancestor of the marine PS-subclades (marine *Synechococcus *and *Prochlorococcus*) whereas the *Cyanobium*-clade and *Paulinella *are plesiomorphic. Pair 869/908 shows parallel changes in a few other cyanobacteria, (e.g. in *Fischerella*).

Phylogenetic analyses revealed basal cyanobacterial branches (e.g. *Gloeobacter*), and two moderately supported lineages, one combining the majority of the β-cyanobacteria including all plastids (branch 1 in Figure 1), the other containing *Paulinella *and the PS-clade nested within a radiation of a few β-cyanobacteria, representing the clades PHOR, PRCHX and SELONG (branch 2 in Figure 1). *Paulinella *is monophyletic with the PS-clade (branch 4). Our previous study [1] had already revealed the monophyly of both marine PS-subclades to the exclusion of the *Paulinella *chromatophore, as confirmed here (see branch 9). The present investigation includes five sequences of the third PS-subclade, the *Cyanobium*-clade, which is sister to the marine subclades, with *Paulinella *still diverging in a basal position. The monophyly of the entire PS-clade to the exclusion of *Paulinella*, however, receives only moderate to low bootstrap support (branch 5), but is corroborated by unique synapomorphies in the 23S rRNA (Figure 2). As previously shown, both marine PS-subclades are characterized by unique compensatory base changes (CBCs in pairs 868/909 and 869/908), whereas *Paulinella *is plesiomorphic in both pairs [1]. Interestingly, the *Cyanobium*-clade is intermediate in sharing the unique CBC in position 868/909 with marine PS-subclades, but displaying the ancestral character state in positions 869/908, in congruence with the tree topology (Figure 2).

Several cyanobacterial branches gained only moderate bootstrap support (e.g. branches 1, 2, 3, 5, 12 in Figure 1). One possible explanation is the base compositional bias among prokaryotic rDNAs (the Chi-square test gave p = 0.00) mainly caused by bacteria and plastids (cyanobacteria alone have no significant base compositional bias; p = 0.21). Therefore we performed distance analyses using the LogDet correction for unequal base composition (Additional File 1) that largely confirm results shown in Figure 1. Branch 5 (PS-clade without *Paulinella*) is even better supported (78%). The phylogenetic signal for branch 5 is confined to the 23S rRNA gene as shown by single-gene analyses (87–93% bootstrap in the 23S rRNA phylogeny), since this branch collapsed in 16S rDNA analyses, as also did the branches 9, 11, 12, and 14 (Additional File 1).

In conclusion, the rDNA data support monophyly of both marine PS-subclades (= α-cyanobacteria sensu [11] with proteobacterial form 1A RubisCO) to the exclusion of both the *Cyanobium*-clade and the *Paulinella *chromatophore, raising the question whether these taxa are affiliated with α- or with β-cyanobacteria (form 1B RubisCO), i.e. on which branch of the phylogenetic tree the horizontal gene transfer of form 1A RubisCO occurred. To answer this question, sequencing and phylogenetic analysis of RubisCO of the *Cyanobium*-clade and the *Paulinella *chromatophore was required.

### Horizontal gene transfer of a carboxysomal operon into the ancestor of the Paulinella chromatophore

**Figure 3:**
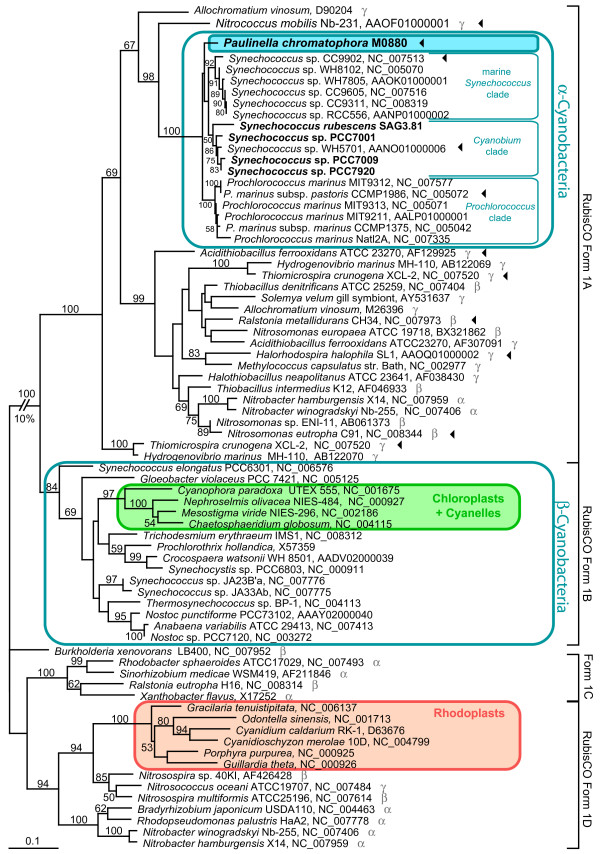
**Evidence for HGT of RubisCO form 1A to the common ancestor of the *Paulinella *chromatophore and α-cyanobacteria**. The ML tree was inferred from RubisCO large subunit (*rbcL*) form 1 amino acid sequences (470 aligned positions) of *Paulinella chromatophora*, cyanobacteria, plastids and proteobacteria under the RtREV+I+Γ model of amino acid substitution. Numbers at branches are ML bootstrap values ≥ 50%. Strain designations (when available) and NCBI accession numbers are indicated after the species name. Newly determined sequences are given in bold. Greek letters in grey indicate α-, β-, or γ- proteobacteria. Arrowheads highlight strains for which the gene arrangement of the carboxysomal operon is shown in Figure 5.

In this study complete *rbcL *(RubisCO large subunit) sequences of *Paulinella *and four members of the *Cyanobium*-clade were determined and integrated into a global alignment of form 1 RubisCO large subunit amino acid sequences. The phylogenetic analysis resolves *Paulinella chromatophora *and the *Cyanobium*-clade as monophyletic with marine *Synechococcus *and *Prochlorococcus *spp., in congruence with the rDNA phylogeny (Figure 3). The *Paulinella *chromatophore as well as the entire PS-clade belong to the RubisCO form 1A lineage, and in conclusion, have to be considered as α-cyanobacteria. The position of the entire α-cyanobacterial clade (including *Paulinella*) in the *rbcL *tree is not congruent with the rDNA phylogeny: α-cyanobacteria are not monophyletic with the remaining cyanobacteria (β-cyanobacteria) but are nested within a radiation of α-, β- and γ-proteobacteria in the *rbcL *phylogeny (Figure 3). This incongruence reflects the horizontal gene transfer (HGT) of RubisCO form 1A from proteobacteria to α-cyanobacteria, and loss of the ancestral 'cyanobacterial' RubisCO form 1B [9].

In cyanobacteria, carboxysomes are essential for the carbon concentration mechanism (CCM; [19]). Physiological differences between form 1A and form 1B RubisCO and corresponding carboxysome types (α and β) are still not understood [19,20]. It has been suspected that the occurrence of α-carboxysomes is correlated with ecological restriction to marine open ocean habitats [19]. Clearly, this view is untenable, since the *Cyanobium*-clade, which predominantly contains freshwater species, as well as the *Paulinella *chromatophore also display α-carboxysomes. Since α-cyanobacteria occur in a broad range of habitats, it is even more difficult to speculate about advantages of α-carboxysomes for survival in special ecological niches, in particular an endosymbiotic habitat (*Paulinella*). As the microenvironment of the chromatophore of *Paulinella *can presumably be characterized as CO_2_-rich due to host respiration, it may even be assumed that an efficient CCM may not be essential for the *Paulinella *chromatophore. Regrettably, experimental data on the photosynthetic properties of the *Paulinella *chromatophore, especially the existence and effectiveness of a CCM, are not yet available.

**Figure 4:**
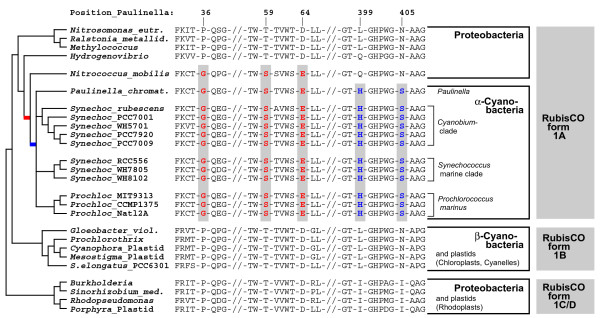
**Unique synapomorphies highlighting the HGT of RubisCO from a *Nitrococcus*-like γ-proteobacterium to the common ancestor of the *Paulinella*  chromatophore and free-living α-cyanobacteria**. Selected regions of the RubisCO large subunit amino acid sequence were plotted against a simplified phylogram (NJ bootstrap consensus tree). Three synapomorphies shared by *Nitrococcus *and α-cyanobacteria are shown in red colour (positions 36, 59, 64); Histidine 399 and Serine 405 (blue) are unique for *Paulinella *and free-living α-cyanobacteria to the exclusion of all proteobacterial ancestors and β-cyanobacteria.

Previous *rbcL *phylogenies did not contain the γ-proteobacterium *Nitrococcus mobilis *(genome sequence available since Feb-2006) [10,11,20,21]. Interestingly, our *rbcL*-phylogeny identified this taxon as closest relative to the α-cyanobacteria: the common branch of *Nitrococcus *and the α-cyanobacteria gained 98% bootstrap support (Figure 3). A search for unique synapomorphies in *rbcL *amino acid sequences revealed 3 positions (AA 36, 59, 64 in the *Paulinella *sequence), which in *Nitrococcus *and all α-cyanobacteria share uniquely derived character states to the exclusion of all remaining proteobacterial and β-cyanobacterial sequences (Figure 4). In addition, we found two synapomorphies (positions 399, 405), which characterize the α-cyanobacterial form 1A RubisCO (Figure 4). These results highlight *Nitrococcus *as a key taxon for the HGT of RubisCO form 1A, being the closest known relative of the proteobacterial donor of *rbcL*.

Another HGT event is responsible for the well-known polyphyly of plastids in *rbcL *phylogenies [9,21,22]. Chloroplasts and cyanelles are rooted in the β-cyanobacteria in congruence with ribosomal phylogenies (Figure 1). In contrast, rhodoplasts are nested within the RubisCO form 1D clade of α-, β- and γ-proteobacteria. With high significance, the analysis reveals *Nitrosospira sp., Nitrosococcus oceani *and *Nitrosospira multiformis *as a sister branch to rhodoplasts (the latter two already described in [21].)

**Figure 5:**
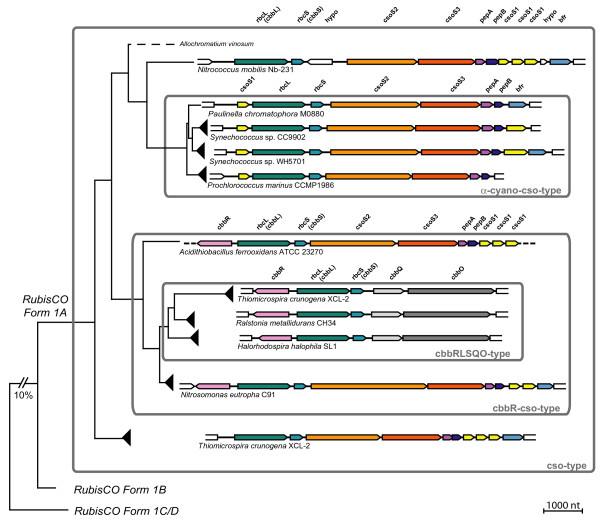
**Architecture and evolution of operons containing form 1A RubisCO from proteobacteria and α-cyanobacteria including the *Paulinella *chromatophore**. Gene arrangements from selected taxa (see arrowheads in Figure 3) are plotted against a simplified phylogram based on RubisCO amino acid sequences. Four major types of gene arrangements can be distinguished (for details, see text). The operon of *Paulinella *is member of the α-cyano-cso-type, which is derived from the ancestral cso-type present in proteobacteria, providing evidence for a HGT of the complete operon. Homologous genes share the same colour. Abbreviations: Carboxysomal shell proteins 1, 2, 3 (csoS1, 2, 3); RubisCO large and small subunit (*rbcL*, *rbcS *= *cbbL*, *cbbS*); carboxysomal peptides A, B (*pepA*, *pepB*); bacterioferritin (*bfr*); LysR-type transcriptional activator (*cbbR*); putative RubisCO activation proteins (*cbbQ*, *cbbO*); hypothetical proteins (hypo). Dotted lines indicate that no data are available.

In the α-cyanobacteria, the genes encoding RubisCO form 1A belong to an operon that further contains genes for carboxysomal proteins, and previous studies revealed that the complete carboxysomal operon was acquired by HGT [10,11]. Among proteobacteria gene content as well as gene order in *rbcL*-containing operons differs considerably [23]. Several proteobacteria have two or even three unrelated *rbcL *genes (see for example *Nitrobacter*, *Hydrogenovibrio*, *Thiomicrospira *in Figure 3; [23,24]). In contrast, cyanobacteria generally have only one *rbcL *gene. In the present study, we determined the sequence of the complete carboxysomal operon of *Paulinella chromatophora *(7.6 kb), and compared the gene arrangement among members of the RubisCO form 1A clade (Figure 5).

We found four major arrangement types. To analyse the evolution of these types, we plotted operon structures against a simplified *rbcL *tree as shown in Figure 5 (for more details, see Additional File 2). Basal branches of the RubisCO form 1A radiation (e.g. *Thiomicrospira*, *Nitrococcus*) show an almost identical operon architecture, which likely represents the plesiomorphic state. In this type, (cso-type) the following genes occur downstream of *rbcL*: *rbcS*, *csoS2*, *csoS3*, *pepA*, *pepB*, *csoS1*, *csoS1*, *csoS1*, and the iron storage protein bacterioferritin (*bfr*) [25]. The operon in the α-cyanobacteria (α-cyano-cso-type) was derived from the ancestral state by transfer of a single *csoS1 *gene to the 5' end of the operon, accompanied by a reduction of the number of 3' located *csoS1 *copies to one or zero. Interestingly, two members of the α-cyanobacteria still contain the 3' bacterioferritin gene (*bfr*): the *Paulinella *chromatophore and *Synechococcus *WH5701, the latter representing the *Cyanobium*-clade (data for the remaining *Cyanobium*-clade are currently missing). In the marine PS-subclades, *bfr *is absent (Figure 5; Additional File 2). Comparison with the ribosomal phylogeny (Figure 1) suggests that bacterioferritin was acquired by the same HGT event as the carboxysomal operon, and was secondarily lost in marine *Synechococcus *and *Prochlorococcus *species. We addressed the *bfr *HGT hypothesis by performing a phylogenetic analysis with α- and β-cyanobacterial ferritins and their proteobacterial relatives (Additional File 3). As in the *rbcL*-phylogeny, the bacterioferritins of *Paulinella *and WH5701 are monophyletic with *Nitrococcus mobilis *as their closest relative, and were nested within proteobacteria with carboxysomal operons, clearly proving co-transfer of bacterioferritin with the carboxysomal operon. Neither nonheme-ferritins of marine *Synechococcus*/*Prochlorococcus*-species nor the ferritin genes of β-cyanobacteria show any relationship to *bfr *of *Paulinella *and WH5701 (for details, see Additional File 3). In γ-proteobacteria incl. *Nitrococcus*, and in *Prochlorococcus*, the next gene downstream to the carboxysomal operon is a putative pterine-4alpha-carbinolamine dehydratase (Additional File 2) that in the remaining α-cyanobacteria is also present, but in those taxa is not linked to the carboxysomal operon. Blast searches [26] reveal their homology, suggesting that besides the carboxysomal operon and *bfr *even more genes may have been acquired by the same HGT event.

In parallel to the α-cyano-cso-type, another proteobacterial gene arrangement type is derived from the cso-type by the acquisition of a *cbbR *gene upstream to *rbcL*, coded by the opposite strand (Figure 5). This type is therefore here named cbbR-cso-type (e.g. *Nitrosomonas eutropha*, Figure 5). Though not co-transcribed with the carboxysomal genes, CbbR is linked to this operon by its specific function as a transcriptional activator [27]. Finally, the most derived gene arrangement type is nested within the cbbR-cso-type, and is named cbbRLSQO-type. The first three genes, *cbbR*, *rbcL*, and *rbcS*, remained unchanged whereas all carboxysomal shell proteins and bacterioferritin were lost and replaced by the genes *cbbQ *and *cbbO*, which are absent in the remaining three types of operon structures (Figure 4). *cbbQ *and *cbbO *have been shown to enhance RubisCO activity and stability [28,29].

Notably, each synapomorphic change leading to the three evolutionary derived gene arrangement types corresponds to a single branch/clade in the phylogenetic tree based on *rbcL *sequence data (Figure 3). This congruence provides additional credibility for the *rbcL *tree, including one branch without any bootstrap support that combines all taxa characterized by the cbbRLSQO-type (Figures 4, 5). However, the loss of bacterioferritin in the marine *Synechococcus *and *Prochlorococcus *clades, which can also be assumed to be a synapomorphic change, cannot be traced to a single branch in the *rbcL *tree due to low resolution among α-cyanobacteria. Although the four major lineages (*Paulinella*, *Cyanobium*-clade, marine *Synechococcus*- and *Prochlorococcus*-clades) are recovered, relationships between these lineages remain unresolved (Figure 3). Based on the rDNA phylogeny, it appears likely that bacterioferritin was uniquely lost in the common ancestor of marine α-cyanobacterial clades as a single synapomorphic change (branch 9 in Figure 1). In general, the branching order within the α-cyanobacteria shows no significantly supported conflict between rDNA and *rbcL *phylogenies, and thus, both data sets were used separately and in combination to resolve phylogenetic relationships within the α-cyanobacteria.

### Phylogenetic resolution of the Synechococcus/Prochlorococcus-clade with the concatenated dataset

**Figure 6:**
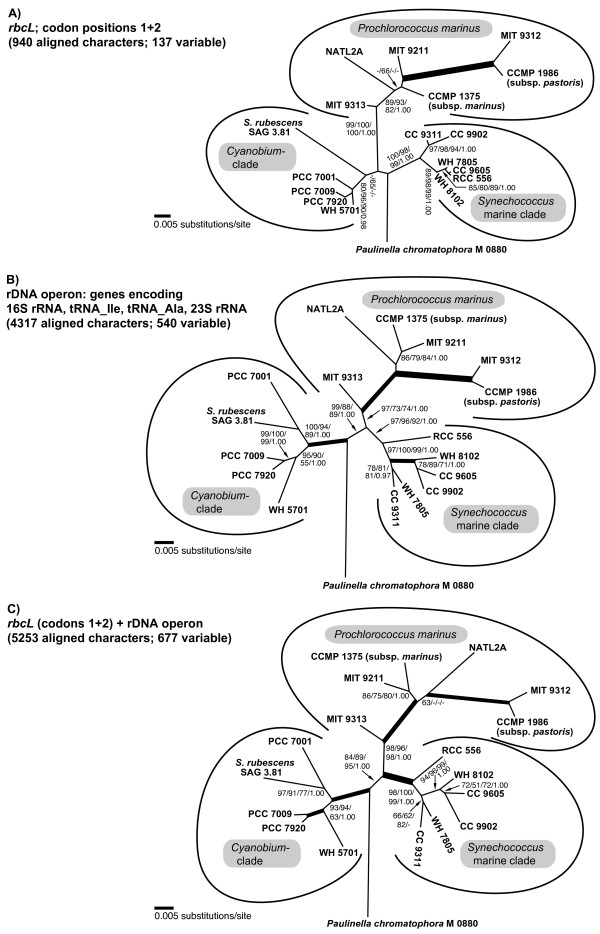
**Comparison of phylogenetic relationships among *Paulinella *and free-living α-cyanobacteria inferred by *rbcL *and/or rDNA nucleotide sequence data**. **A**. Unrooted analysis of codon positions 1+2 of the *rbcL *gene. **B**. Phylogeny of the rDNA operon, using more aligned positions as in Figure 1 (4317 vs. 4126 characters). **C**. Phylogeny inferred from concatenated *rbcL *and rDNA sequences. Tree topologies resulted from ML analyses using a GTR+I+Γ model; significance values shown as in Figure 1.

**Figure 7:**
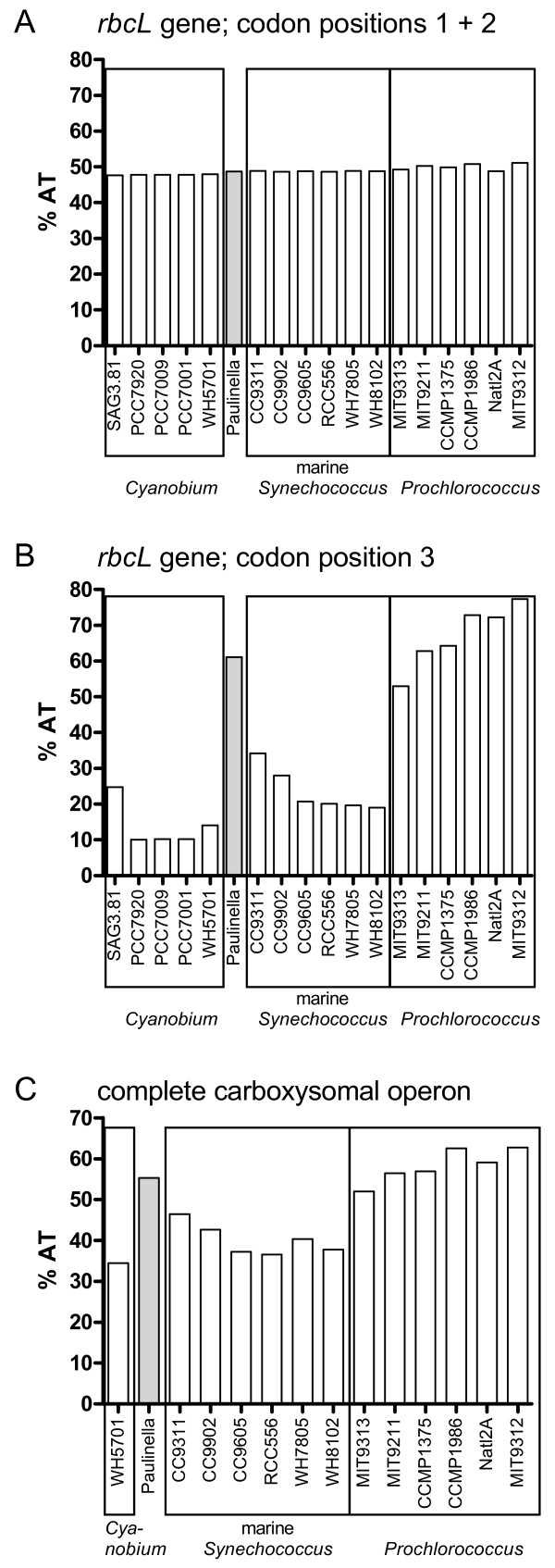
**AT content bias in *rbcL *and complete carboxysomal operon sequences in *Paulinella *and *Prochlorococcus***. Whereas the AT-content in codon positions 1 + 2 in the *rbcL *gene is balanced across *Paulinella *and 17 free-living α-cyanobacteria (**A**), the third codon position displays a sharply elevated AT-content in *Paulinella *as well as *Prochlorococcus *strains (**B**). The AT-content integrated over the complete carboxysomal operon (from *csoS1 *to *pepB*) including intergenic spacer regions shows a similar bias, although less pronounced (**C**).

In Figure 6, three phylogenetic analyses of *P. chromatophora *and 17 taxa of *Synechococcus *and *Prochlorococcus *using three different datasets are compared: (A) *rbcL *nucleotide sequences, (B) complete rDNA operon sequences, and (C) concatenated *rbcL *and rDNA sequences. Prior to phylogenetic analyses, nucleotide frequencies of *rbcL *and rDNA sequence data were determined to prevent artefacts caused by base compositional bias. Whereas the base composition of *rbcL *codon positions one and two was homogenous across taxa (Figure 7A), the third codon position revealed strong differences between AT-rich (*Paulinella*, and *Prochlorococcus *strains: ca. 50–80%) and AT-poor taxa (*Synechococcus *strains ca. 10–30%) (Figure 7B). Therefore, only first and second codon positions were used for phylogenetic analyses of the *rbcL *gene.

Both *rbcL *and rDNA phylogenies recover the monophyly of three major clades: *Cyanobium*-clade, marine *Synechococcus*-clade and *Prochlorococcus*-clade. However, *rbcL *data fail to recover the *Cyanobium*-clade with significance, probably due to the long-branched *Synechococcus rubescens *(see also the amino acid analysis; Figure 3). On the other hand, *Prochlorococcus *displays a much longer basal branch in the *rbcL *analysis compared to rDNA, due to a higher number of synapomorphies, accompanied by a considerably higher bootstrap support (99–100% in *rbcL *compared to 73–97% in rDNA). The higher-level order of the major clades remains unresolved in the *rbcL *tree, whereas the rDNA analysis recovers the sister-group relationship of marine *Synechococcus *and Prochlorococcus clades (88–99% bootstrap; see also Figure 1). Within *Prochlorococcus*, relationships among strains are resolved almost congruently by *rbcL *and rDNA data: MIT9313 appears as the basal divergence, followed by strains NATL2A, CCMP1375 and MIT9213 (relationships among these strains are not resolved), and a derived, long-branched clade consisting of MIT9312 and CCMP1986. In the remaining clades (*Cyanobium*-clade and marine *Synechococcus*-clade), the branching pattern of most strains remains unresolved by *rbcL *analyses, due to their almost redundant *rbcL *sequences. In contrast, the higher rDNA sequence diversity allows a better resolution in these lineages. For instance, *Synechococcus rubescens*, a single long branch in the *rbcL *tree, is confidently identified as sister of PCC 7001 by rDNA data. Similarly, *Synechococcus *strain RCC 556, which has an almost identical *rbcL *sequence as three other strains (CC9605, WH7805, WH8102), is resolved as the basal divergence of the marine *Synechococcus *clade by rDNA analyses. The concatenated data set (5253 nucleotide characters) is largely congruent with the rDNA tree, albeit with even better bootstrap support for the monophyly of the major clades (especially *Prochlorococcus *and marine *Synechococcus*). However, in a few cases (e.g. position of *Synechococcus rubescens*, position of *Synechococcus *CC9311) the combined analysis showed slightly reduced support in comparison with the rDNA phylogeny (Figure 6B, 6C).

In the phylogenetic analyses performed in this study, we found no specific and robustly supported relationship between *Paulinella *and any of the three major clades of free-living α-cyanobacteria. *RbcL *data fail to resolve the monophyly of the *Cyanobium*-clade to the exclusion of *Paulinella *with significance, irrespective of whether amino acid or nucleotide data are analyzed (Figures 3, 6A), in contrast to the remaining phylogenies that receive high significance for this clade (Figures 1, 6B, 6C). Although the prokaryote-wide rDNA phylogeny (Figure 1) uses fewer positions than the unrooted rDNA phylogeny of the α-cyanobacteria (Figure 6B; 4126 vs. 4317 characters), we recognize almost complete congruence between the two phylogenies among α-cyanobacteria. In addition, our tree topologies corresponded almost completely to phylogenetic analyses of the 16S-23S rDNA spacer ('ITS'; 233 aligned positions) from 57 free-living α-cyanobacteria (Figure 4A in [30]). Probably due to the much higher number of aligned/variable (4317/540) positions in the rRNA and tRNA genes, our analyses gained high significance values for most basal and internal branches that were largely non-significant in ITS-phylogenies [30], e.g. the branch uniting all *Prochlorococcus *strains including the basal divergence MIT9313. Other phylogenies that included only a few α-cyanobacteria had often failed to position strain MIT9313 as monophyletic with other *Prochlorococcus *strains (e.g. [1,31,32]), even in multigene analyses of complete cyanobacterial genomes [31,32]. Superiority of a 233-character-analysis over whole-genome phylogenies may highlight the importance of a sufficient taxon sampling. Moreover, phylogenetic information content of protein gene data may be impaired by unequal base composition and amino acid frequencies across taxa: whereas derived *Prochlorococcus *strains have a high AT-content and show a preference for amino acids encoded by AT-rich codons, base composition and amino acid usage of *Prochlorococcus *MIT9313 are more similar to *Synechococcus *strains with lower AT-content [33], explaining artificial tree topologies. In the more conserved *rbcL *gene, the AT bias is confined to the third codon positions (Figure 7A, 7B), and thus, phylogenies using only the first and second positions are not affected by base compositional bias. In fact, our *rbcL *tree does not contradict analyses using rDNA data, which are known to have a more balanced base composition across taxa ([33], and our own results). Interestingly, *Paulinella *shows the same tendency towards high AT-content as known for *Prochlorococcus*, as evident from third *rbcL *codon positions (Figure 7B) as well as the complete carboxysome operon (Figure 7C). An elevated AT content, accompanied by genome size reduction, is a highly unusual phenomenon for free-living organisms, and in the case of *Prochlorococcus*, was interpreted as adaptation to oligotrophic marine environments with low nitrogen and phosphorus availability [33]. In contrast, endosymbionts or intracellular pathogens are known to tend to evolve towards AT rich genomes (e.g. [34-37]). We suspect that the complete genome of the *Paulinella *chromatophore is characterized by high AT content since not only the carboxysomal operon (this study) but also two DNA fragments of 9.4 kb and 4.3 kb (see Figure 1 in [6]) support this view. Together with its isolated position in molecular phylogenies ([1,6], and this study), the elevated AT content further indicates that the chromatophore of *Paulinella *has significantly diverged from its free-living ancestor, and undergone typical steps in the evolution of an intracellular symbiont such as genome reduction.

## Conclusion

The basal divergence of the *Paulinella *chromatophore as sister to free-living α-cyanobacteria was revealed by phylogenetic analysis of the complete rDNA operon with an extended taxon sampling, especially by addition of the *Cyanobium*-clade. *Paulinella *and free living α-cyanobacteria share a proteobacterial carboxysomal operon with a form 1A RubisCO, indicating that the HGT of the carboxysomal operon predated the divergence of the *Paulinella *chromatophore. The γ-proteobacterium *Nitrococcus mobilis *was identified as the closest known relative to the proteobacterial donor of the carboxysomal operon. The isolated position of *Paulinella *among α-cyanobacteria in molecular phylogenies as well as the elevated AT content of several of its genes indicates that *Paulinella *has already undergone typical steps in reductive genome evolution associated with an intracellular lifestyle.

## Methods

### Algal cultures

Strains used in this study were obtained from the following sources: *Spirulina *sp. PCC 6313, *Microcoleus *sp. PCC 7420, *Scytonema *sp. PCC7110, *Pseudoanabaena *sp. PCC 7367, *Pseudoanabaena *sp. PCC 6903, *Synechococcus *sp. PCC 7001, *Synechococcus *sp. PCC 7920, *Synechococcus *sp. PCC 7009: Pasteur Culture Collection of Cyanobacteria, Institute Pasteur, Paris, France [38]; *Fischerella muscicola *SAG 2027, *Prochlorothrix hollandica *SAG 10.89, *Chroococcidiopsis *sp. SAG 2025, *Synechococcus rubescens *SAG 3.81: Sammlung für Algenkulturen, University of Göttingen, Germany [39]. *Paulinella chromatophora *M0880: Culture collection Melkonian, University of Cologne, Germany;*Cryptomonas curvata *CCAC 0006: Culture Collection of Algae at the University of Cologne, Germany [40].

### DNA extraction, PCR and sequencing

Complete genomic DNA was extracted using a CTAB protocol. PCR primers for amplification of the rDNA operon, and sequencing methods were described previously [1]. For amplification of the full length *rbcL *gene from *Paulinella chromatophora *and *Synechococcus *strains, α-cyanobacteria-specific primers were designed using an alignment of cyanobacterial *rbcL *and surrounding genes. PCR primers bind in the neighbouring genes of *rbcL*: *csoS1 *and *rbcS*. Primer sequences: Al_csoS1_F2: (GARGCWGCWGAYGCHATGACCAAGG) and Al_rbcS_R1: (TGRTCGTADATYTCKTCCTGGGTCATMGG). If primary products were too weak a reamplification was done with Al_csos1_F3 (GCHGAAGTKCGYCTKATYGGTCGTG) or Al_csos1_F4 (CGYCCYCAYMGNGAAGTKGAGCCWGC) and Al_rbcS_R1. Using the same alignment new sequencing primers were designed: Al_csoS1_F4 (sequence see above), Al_rbcL_F1 (TTYGARTTYGTHGCBGAAGC), Al_rbcL_R1 (GGCATRTGCCANACRTGRATRCC), Al_rbcL_R2 (ARYTTHGGYTTRATRGTRCARCC). *RbcL *PCR products were purified using the QIAquick PCR Purification Kit (Qiagen) and sequenced by the cycle sequencing method using an ABI 3730 sequencer.

### Alignments and phylogenetic analyses

Newly obtained sequences were combined with database sequences to construct amino acid and nucleotide sequence alignments. Accession numbers of new sequences are AM709625 – AM709637 (rRNA operon), AM701774 – AM701778 (*rbcL*), and EF589049 (carboxysomal operon of *Paulinella*); accession numbers of database sequences are given in Figures 1 and 3. RbcL and ferritin protein alignments were obtained using clustalW, and refined manually; rDNA operon alignments were constructed manually, guided by rRNA and tRNA secondary structure. Unalignable positions were excluded from datasets prior to phylogenetic analyses. *RbcL *nucleotide sequences were aligned according to the RubisCO amino acid alignment. The rDNA-operon analyses contained 4126 aligned characters for prokaryotes (Figure 1), and 4317 for α-cyanobacteria (Figure 6B, 6C); the RubisCO large subunit amino acid dataset contained 470 aligned positions (Figure 3). The α-cyanobacterial *rbcL *nucleotide dataset was reduced to first and second codon position resulting in 940 aligned nucleotides (Figure 6A, 6C). Nucleotide sequence analyses used PAUP 4.0b10, MrBayes_3.1.1, and MODELTEST, as previously described [1]. The homogeneity of base frequencies across taxa was investigated by Chi-square tests (PAUP 4.0b10). When distance analyses used the LogDet+I model, the I-value was copied from the GTR+I+Γ model parameters that were estimated by MODELTEST for the same dataset. ML bootstrap analyses in Figure 1 were constrained towards 3000 rearrangements per replicate. For Bayesian analyses, two MCMC chains with 500000 generations were performed, and the 'burnin' determined by the convergence criterion (see [1]). The search procedure to find molecular characters, which represent unique synapomorphies for a clade of interest, has been described previously [1], and was here applied to nucleotide as well as protein alignments. Protein datasets were subjected to maximum likelihood bootstrap (n = 1000) analyses with Phyml V2.4.5 [41]. The evolutionary model fitting best (RtREV+I+Γ) was determined using ProtTest 1.3 [42] according to the Akaike Information Criterion. Proportion of invariable sites and Γ-shape parameter were calculated in Phyml.

### Carboxysomal operon data

Sequence data of the carboxysomal operon of the *Paulinella *chromatophores were taken from the ongoing *Paulinella *chromatophore genome project. DNA was extracted from crude chromatophore fractions obtained by density centrifugation on a percoll gradient. This DNA was used to generate a small insert library. Random sequencing of 400 clones yielded raw reads overlapping the previously obtained *rbcL *sequence. Using a combination of primer walks and PCR all gaps in the *rbcL *region were closed.

## Authors' contributions

BM determined 13 rDNA operon sequences, and was responsible for rDNA alignments and analyses, constructed the ferritin alignment, and performed synapomorphy searches, leading to Figures 1, 2, 4, 6B, 6C, Additional Files 1, and 3. ECMN isolated chromatophores of *P. chromatophora *for the ongoing genome project, determined 5 *rbcL *sequences, was responsible for RubisCO alignments and analyses, and investigated gene arrangement types and base composition of carboxysomal operons, leading to Figures 3, 5, 6A, 6C, 7, and Additional File 2. BM and ECMN wrote the manuscript. GG determined the sequence of the carboxysomal operon of *P. chromatophora*. MM conceived the study, contributed to its design, and critically revised the manuscript. All authors read and approved the final manuscript.

## Supplementary Material

Additional File 1 **Significance measures for 14 selected clades (encircled numbers in **Figure 1**) in single-gene and combined analyses of rRNA genes**. Analyses used 1000 bootstrap replicates of NJ (LogDet+I model), NJ (GTR+I+Γ model), and MP, and Bayesian posterior probabilities. Numbers of aligned (ch) and variable characters (var) are given for each partition. Click here for file

Additional File 2 **Gene arrangement of operons containing form 1A RubisCO for all strains included in **Figure 3. The table describes the typical gene arrangements in the 4 different arrangement types, defined in Figure 5, and indicates presence (x) or absence () of the gene in a specific species. Abbreviations as given in Figure 5; further abbreviations: n.d.: no data available; ham1: Ham1 like protein; ndhF3: NADH dehydrogenase subunit L; PCD_DCoH: possible pterin-4alpha-carbinolamine dehydratase; GlnK: nitrogen regulatory protein P-II; GGPS: Glucosylglycerol-phosphate synthase; Transp: transposase, mutator type; REC: response regulator receiver protein; Rpe: Ribulose-phosphate 3-epimerase; TktA: Transketolase; DedA: Uncharacterized membrane-associated protein; CBD_II: Cellulose binding domain protein. chlN: light-independent protochlorophyllide reductase subunit N. Click here for file

Additional File 3 **Evidence that bacterioferritin in *Paulinella *and *Synechococcus *WH5701 was acquired by HGT from a *Nitrococcus *-like γ-proteobacterium**. Phylogeny of three ferritin-families occuring in cyanobacteria (α-cyanobacteria in blue; β-cyanobacteria in orange colour) together with their proteobacterial relatives: Bacterioferritin, Nonheme-Ferritin, and Ferritin and Dps ("DNA-binding protein from starved cells"). The ML analysis was performed as in Figure 3, and used 159 aligned amino acid positions. The chromatophore of *Paulinella *and *Synechococcus *WH5701 (representing the *Cyanobium*-clade) are the only cyanobacteria, which possess bacterioferritin linked to the carboxysomal operon (see Figure 5). Similar to the RubisCO phylogeny, *Nitrococcus mobilis *is sister to these taxa in the bacterioferritin clade. Note that many taxa contain more than one ferritin (up to five in *Synechococcus *CC9311), e.g. *Nitrococcus *displays two unrelated bacterioferritin genes, and *Synechococcus *WH5701 has 1 bacterioferritin, one nonheme-ferritin, and one member of the Ferritin and Dps family (indicated by numbers in curly braces). Cyanobacterial ferritins are dispersed into several separate branches, usually nested within bacterial divergences, suggesting many independent HGT events. One to five (in CC9311) nonheme-ferritin genes are characteristic for members of all PS-subclades (presence in the *Paulinella *chromatophore is currently unknown), and their tree topology indicates an early gene duplication followed by later duplication/gene loss events. Click here for file
