# Supplementary material for: The ancestor of the Paulinella chromatophore obtained a carboxysomal operon by horizontal gene transfer from a Nitrococcus-like γ-proteobacterium
Source: BMC Evol Biol. 2007 Jun 5;7:85. doi: 10.1186/1471-2148-7-85 (PMC1904183; doi:10.1186/1471-2148-7-85)
Supplement: Additional File 1 — Significance measures for 14 selected clades (encircled numbers in Figure 1) in single-gene and combined analyses of rRNA genes. Analyses used 1000 bootstrap replicates of NJ (LogDet+I model), NJ (GTR+I+Γ model), and MP, and Bayesian posterior probabilities. Numbers of aligned (ch) and variable characters (var) are given for each partition. [file 1471-2148-7-85-S1.PDF]

**No**	**clade**	**16S rDNA** (1433 ch/894 var)	**23S rDNA** (2545 ch/1783 var)	**16 + 23S rDNA** (3978 ch/2677 var)
**1**		-/-/-/0.98	-/68/-/1.00	-/84/-/1.00
**2**		88/66/65/0.98	-/-/-/0.96	-/50/-/1.00
**3**	SELONG + PS + *Paulinella*	68/73/69/1.00	-/-/-/-	-/59/62/0.96
**4**	PS + *Paulinella*	100	100	100
**5**	PS without *Paulinella*	-/-/-/-	93/87/91/1.00	78/-/71/1.00
**6**	*Cyanobium*-clade	88/74/-/-	100/100/98/1.00	100/100/94/1.00
**7**	*Synechococcus* (marine)	91/85/58/1.00	66/59/-/1.00	94/94/75/1.00
**8**	*Prochlorococcus*	92/93/68/1.00	-/-/-/0.99	-/81/78/1.00
**9**	*Synech*ococcus (marine) + *Prochlorococcus*	-/-/-/1.00	81/93/84/1.00	95/97/84/1.00
**10**	PSAN	82/82/56/1.00	100	100
**11**	S/P/M	-/-/-/-	65/-/-/1.00	91/83/75/1.00
**12**	OSC	-/-/-/-	-/-/59/-	56/-/73/1.00
**13**	NOST	99/92/95/1.00	100/98/89/1.00	100/100/99/1.00
**14**	CHROO + NOST	-/-/-/-	99/98/84/1.00	98/99/94/1.00
