# Supplementary material for: The ancestor of the Paulinella chromatophore obtained a carboxysomal operon by horizontal gene transfer from a Nitrococcus-like γ-proteobacterium
Source: BMC Evol Biol. 2007 Jun 5;7:85. doi: 10.1186/1471-2148-7-85 (PMC1904183; doi:10.1186/1471-2148-7-85)
Supplement: Additional File 2 — Gene arrangement of operons containing form 1A RubisCO for all strains included in Figure 3. The table describes the typical gene arrangements in the 4 different arrangement types, defined in Figure 5, and indicates presence (x) or absence () of the gene in a specific species. Abbreviations as given in Figure 5; further abbreviations: n.d.: no data available; ham1: Ham1 like protein; ndhF3: NADH dehydrogenase subunit L; PCD_DCoH: possible pterin-4alpha-carbinolamine dehydratase; GlnK: nitrogen regulatory protein P-II; GGPS: Glucosylglycerol-phosphate synthase; Transp: transposase, mutator type; REC: response regulator receiver protein; Rpe: Ribulose-phosphate 3-epimerase; TktA: Transketolase; DedA: Uncharacterized membrane-associated protein; CBD_II: Cellulose binding domain protein. chlN: light-independent protochlorophyllide reductase subunit N. [file 1471-2148-7-85-S2.PDF]

Type	Organism	Strain		5'	*rbcL*	*rbcS*	*csoS2*	*csoS3*	*pepA*	*pepB*	*csoS1*	*csoS1*	*csoS1*	*bfr*	3'
cso	*N. mobilis*	Nb-231		hypo	x	x	x	x	x	x	x	x	x	x	PCD_DCoH
	A. vinosum			n.d.	x	x	n.d.								
	T. crunogena	XCL-2		hypo	x	x	x	x	x	x	x	x	x	x	PCD_DCoH
	H. marinus	MH-110		n.d.	x	x	x	x	x	x	x	x	x	x	PCD_DCoH
**Type**	**Organism**	**Strain**	**5'**	***csoS1***	***rbcL***	***rbcS***	***csoS2***	***csoS3***	***pepA***	***pepB***	***csoS1***	***csoS1***	***csoS1***	***bfr***	**3'**
α-cyano-cso	*P. chromatophora*	M0880	ham1	x	x	x	x	x	x	x				x	ndhF3
	Synechococcus sp.	CC9902	ham1	x	x	x	x	x	x	x	x				ndhF3
	Synechococcus sp.	WH8102	ham1	x	x	x	x	x	x	x	x				ndhF3
	Synechococcus sp.	WH7805	ham1	x	x	x	x	x	x	x	x				ndhF3
	Synechococcus sp.	CC9605	ham1	x	x	x	x	x	x	x	x				ndhF3
	Synechococcus sp.	CC9311	ham1	x	x	x	x	x	x	x	x				ndhF3
	Synechococcus sp.	RCC556	ham1	x	x	x	x	x	x	x	x				ndhF3
	Synechococcus sp.	SAG3.81		n.d.	x	x	n.d.								
	Synechococcus sp.	PCC7001		n.d.	x	x	n.d.								
	Synechococcus sp.	WH5701	chlN	x	x	x	x	x	x	x	x			x	ndhF3
	Synechococcus sp.	PCC7009		n.d.	x	x	n.d.								
	Synechococcus sp.	PCC7920		n.d.	x	x	n.d.								
	P. marinus	MIT9312	ham1	x	x	x	x	x	x	x					PCD_DCoH
	P. marinus	CCMP1986	ham1	x	x	x	x	x	x	x					PCD_DCoH
	P. marinus	MIT9313	ham1	x	x	x	x	x	x	x	x				PCD_DCoH
	P. marinus	MIT9211	ham1	x	x	x	x	x	x	x	x				PCD_DCoH
	P. marinus	CCMP1375	ham1	x	x	x	x	x	x	x	x				PCD_DCoH
	P. marinus	NATL2A	ham1	x	x	x	x	x	x	x	x				PCD_DCoH
**Type**	**Organism**	**Strain**	**5'**	***cbbR***	***rbcL***	***rbcS***	***csoS2***	***csoS3***	***pepA***	***pepB***	***csoS1***	***csoS1***	***csoS1***	***bfr***	**3'**
cbbR-cso	*A. ferrooxidans*	ATCC 23270	n.d.	x	x	x	x	x	x	x	x	x	x	n.d.	
	H. neapolitanus	ATCC 23641		n.d.	x	x	x	x	x	x	x	x	x	n.d.	
	T. intermedius	K12		n.d.	x	x	x	x	x	x	x	x	x	n.d.	
	N. hamburgensis	X14	GlnK	x	x	x	x	x	x	x	x	x			PCD_DCoH
	N. winogradskyi	Nb-255	GlnK	x	x	x	x	x	x	x	x	x			PCD_DCoH
	Nitrosomonas sp.	ENI-11		n.d.	x	x	n.d.								

	Nitrosomonas sp.	C91	GlnK	**x**	**x**	**x**	**x**	**x**	**x**	**x**	**x**	**x**		**x**	PCD_DCoH

**Type**	**Organism**	**Strain**	**5'**	***cbbR***	***rbcL***	***rbcS***	***cbbQ***	***cbbO***	**3'**
cbbRLSQO	*H. marinus*	MH-110	n.d.	**x**	**x**	**x**	**x**	**x**	hypo
	T. crunogena	XCL-2	cbbR	**x**	**x**	**x**	**x**	**x**	hypo
	T. denitrificans	ATCC25259	GGPS	**x**	**x**	**x**	**x**	**x**	hypo
	S. velum Symbiont			n.d.	**x**	**x**	**x**	**x**	n.d.
	A. vinosum			n.d.	**x**	**x**	n.d.		
	R. metallidurans	CH34	Transp	**x**	**x**	**x**	**x**	**x**	hypo
	N. europaea	ATCC 19718	REC	**x**	**x**	**x**	**x**	**x**	hypo
	A. ferrooxidans	ATCC 23270		n.d.	**x**	**x**	**x**	n.d.	
	H. halophila	SL1	Rpe	**x**	**x**	**x**	**x**	**x**	TktA
	M. capsulatus	Bath	DedA		**x**	**x**	**x**		CBD_II
