# Supplementary figures and images for: The ancestor of the Paulinella chromatophore obtained a carboxysomal operon by horizontal gene transfer from a Nitrococcus-like γ-proteobacterium
Source: BMC Evol Biol. 2007 Jun 5;7:85. doi: 10.1186/1471-2148-7-85 (PMC1904183; doi:10.1186/1471-2148-7-85)

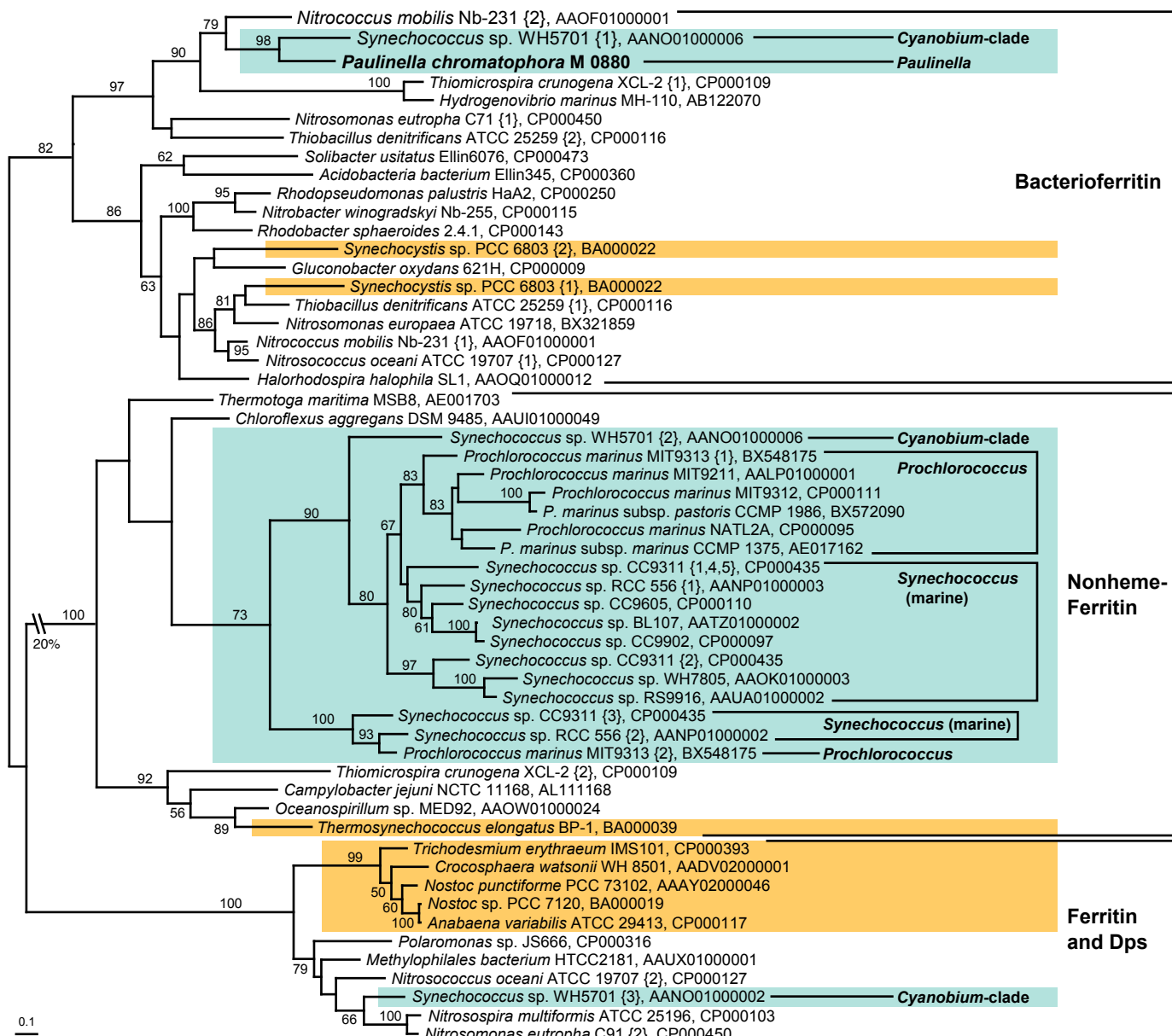

Supplement: Additional File 3 — Evidence that bacterioferritin in Paulinella and Synechococcus WH5701 was acquired by HGT from a Nitrococcus -like γ-proteobacterium. Phylogeny of three ferritin-families occuring in cyanobacteria (α-cyanobacteria in blue; β-cyanobacteria in orange colour) together with their proteobacterial relatives: Bacterioferritin, Nonheme-Ferritin, and Ferritin and Dps ("DNA-binding protein from starved cells"). The ML analysis was performed as in Figure 3, and used 159 aligned amino acid positions. The chromatophore of Paulinella and Synechococcus WH5701 (representing the Cyanobium-clade) are the only cyanobacteria, which possess bacterioferritin linked to the carboxysomal operon (see Figure 5). Similar to the RubisCO phylogeny, Nitrococcus mobilis is sister to these taxa in the bacterioferritin clade. Note that many taxa contain more than one ferritin (up to five in Synechococcus CC9311), e.g. Nitrococcus displays two unrelated bacterioferritin genes, and Synechococcus WH5701 has 1 bacterioferritin, one nonheme-ferritin, and one member of the Ferritin and Dps family (indicated by numbers in curly braces). Cyanobacterial ferritins are dispersed into several separate branches, usually nested within bacterial divergences, suggesting many independent HGT events. One to five (in CC9311) nonheme-ferritin genes are characteristic for members of all PS-subclades (presence in the Paulinella chromatophore is currently unknown), and their tree topology indicates an early gene duplication followed by later duplication/gene loss events. [file 1471-2148-7-85-S3.PDF]
